# Synthesis of renewable high-density fuel with isophorone

**DOI:** 10.1038/s41598-017-06556-7

**Published:** 2017-07-21

**Authors:** Wei Wang, Yanting Liu, Ning Li, Guangyi Li, Wentao Wang, Aiqin Wang, Xiaodong Wang, Tao Zhang

**Affiliations:** 1 Shaanxi Key Laboratory of Catalysis, School of Chemistry and Environment Science, Shaanxi University of Technology, No. 1 Dongyihuan Road, Hanzhong, 723001 China; 2 State Key Laboratory of Catalysis, Dalian Institute of Chemical Physics, Chinese Academy of Sciences, No. 457 Zhongshan Road, Dalian 116023, No. 457 Zhongshan Road, Dalian, 116023 China; 3 iChEM (Collaborative Innovation Centre of Chemistry for Energy Materials), Dalian Institute of Chemical Physics, Chinese Academy of Sciences, No. 457 Zhongshan Road, Dalian, 116023 China

## Abstract

1,1,3-Trimethyl-5-(2,4,4-trimethylcyclohexyl)cyclohexane, a renewable high density fuel, was first produced in a high overall carbon yield (~70%) with isophorone which can be derived from hemicellulose. The synthetic route used this work contains three steps. In the first step, 3,3,5-trimethylcyclohexanone was synthesized by the selective hydrogenation of isophorone. Among the investigated catalysts, the Pd/C exhibited the highest activity and selectivity. Over this catalyst, a high carbon yield (99.0%) of 3,3,5-trimethylcyclohexanone was achieved under mild conditions (298 K, 2 MPa H_2_, 1 h). In the second step, 3,5,5-trimethyl-2-(3,3,5-trimethylcyclohexylidene)cyclohexanone was produced in a high carbon yield (76.4%) by the NaOH catalyzed self-aldol condensation of 3,3,5-trimethylcyclohexanone which was carried out in a round bottom flask attached to the Dean–Stark apparatus. In the third step, the 3,5,5-trimethyl-2-(3,3,5-trimethylcyclohexylidene)cyclohexanone was hydrodeoxygenated under solvent-free conditions. High carbon yield (93.4%) of 1,1,3-trimethyl-5-(2,4,4-trimethylcyclohexyl)cyclohexane was obtained over the Ni/SiO_2_ catalyst. The 1,1,3-trimethyl-5-(2,4,4-trimethylcyclohexyl)cyclohexane as obtained has a density of 0.858 g mL^−1^ and a freezing point of 222.2 K. As a potential application, it can be blended into conventional fuels (such as RP-1, RG-1, *etc*.) for rocket propulsion.

## Introduction

With the increasing of social concern about the sustainable energy and environmental problems, the catalytic conversion of renewable biomass to high quality fuel^1–3^ and useful chemicals^4–9^ has drawn a lot of attention. Polycycloalkanes are a family of nontoxic propellants which are widely used for rockets and missile^10–13^. Due to their relatively higher densities (or volumetric heat values) than traditional refined fuels, polycycloalkanes can be used to increase the range and payload of aircrafts without increasing the volume of fuel tank. This character is especially useful for rocket to save more space (or weight) for electronic equipment, astronauts and other components.

Currently, the most used rocket fuels (such as RP-1, RG-1, *etc*.) are derived from the petroleum in few special oil fields^14^. In the long run, the exploration of new route for synthesis of high-density fuels with the renewable and CO_2_ neutral biomass is highly expected. During the past years, several routes have been developed for the production of polycycloalkanes with terpenes^15–18^. Due to the limited resource of terpenes, it is still necessary to develop some new synthetic route for polycycloalkanes with cheaper and more available biomass^19–26^. Hemicellulose is one of the major components of agriculture and forest wastes (see supplementary Table S1 for the hemicellulose contents in various terrestrial biomasses). Isophorone is the trimerization product of acetone which is the by-product in the Acetone-Butanol-Ethanol fermentation of hemicellulose^27^. Based on the cyclic chemical structure of this compound, we think that it can be used as potential feedstock for the synthesis of high-density polycycloalkanes. To the best of our knowledge, there is no report about this.

**Figure 1:**
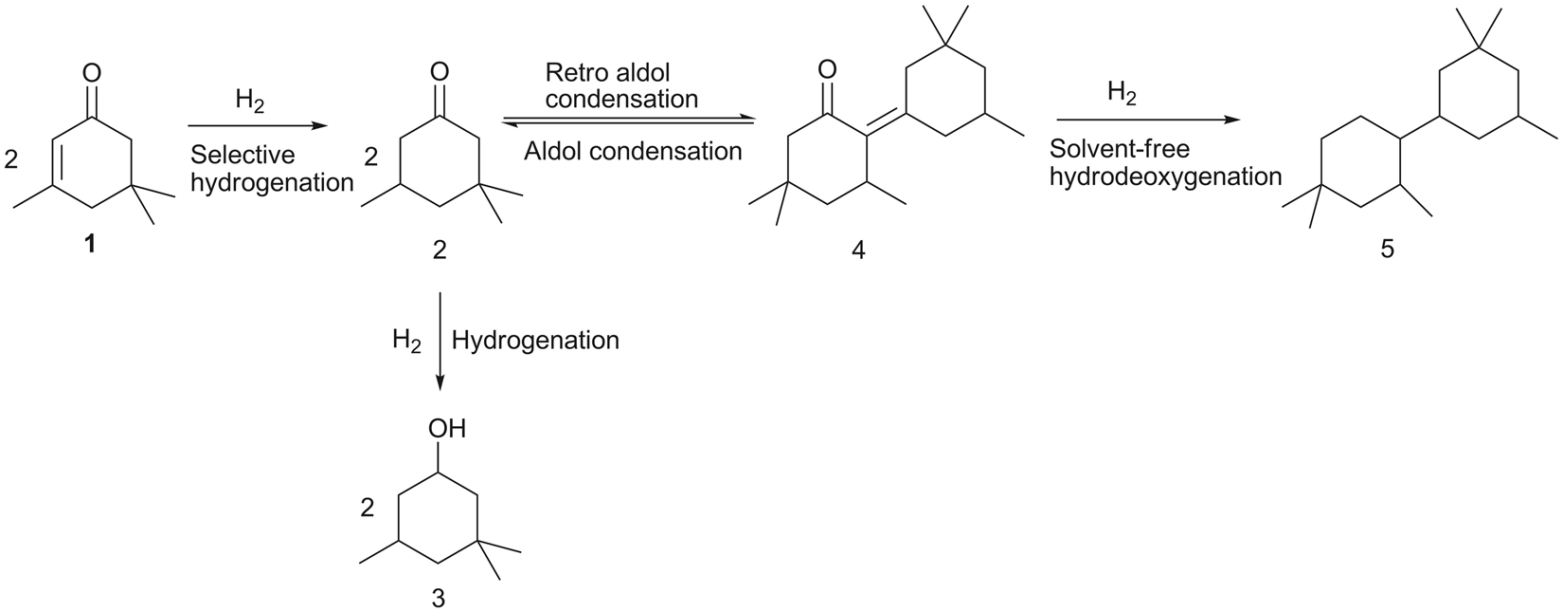
Strategy for the synthesis of compound 2 with isophorone and hydrogen.

In this work, 1,1,3-trimethyl-5-(2,4,4-trimethylcyclohexyl)cyclohexane (*i.e*. the compound **5** in Fig. 1), a C_18_ bicycloalkane with a density of 0.858 g mL^−1^ and a freezing point of 222.2 K, was first synthesized in an overall carbon yield of ~70% by the selective hydrogenation and the self-aldol condensation of isophorone, followed by the solvent-free hydrodeoxygenation (HDO) of the C_18_ condensation product. The synthetic route for this C_18_ bicycloalkane was illustrated in Fig. 1. As a potential application, the compound **5** obtained in this work can be blended into conventional high-density fuels for rocket propulsion.

## Results and Discussion

### Synthesis of 3,3,5-trimethylcyclohexanone

**Figure 2:**
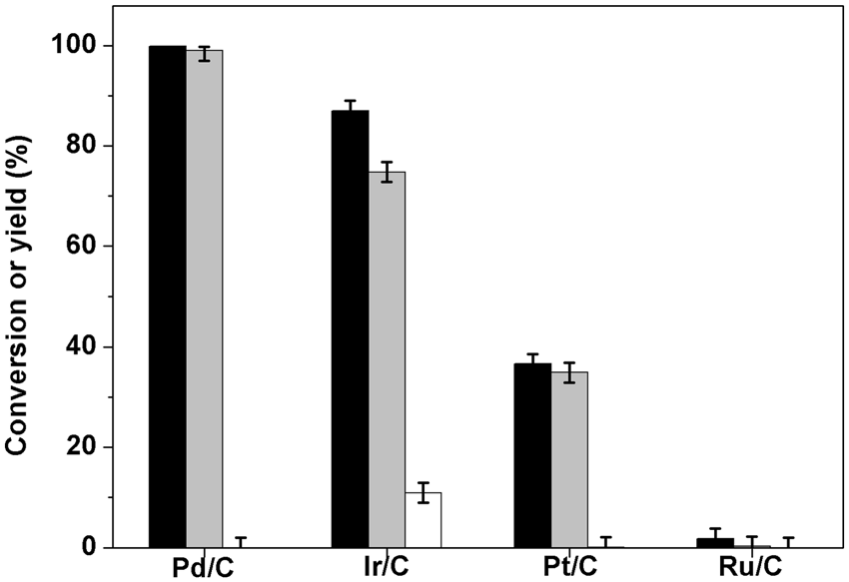
Isophorone conversions (black bars) and the carbon yields of compound 2 (gray bars), and compound 3 (white bars) over the different catalysts. Reaction conditions: 298 K, 1 h; 1.16 g (8.4 mmol) isophorone, 0.05 g catalyst, 2.0 MPa H_2_.

3,3,5-Trimethylcyclohexanone (*i.e*. the compound **2** in Fig. 1) is a chemical which is widely used as a solvent for vinyl resins, laquers, varnishes, paints and other coatings^28, 29^. In the first part of this work, we investigated the selective hydrogenation of isophorone to compound **2** over a series of noble metal catalysts (see Fig. 2). From the analysis of GC and NMR spectra (see Supplementary Figs S1 and S2), compound **2** was identified as the major component in the hydrogenation products. This result can be rationalized because the hydrogenation of C = C bond in isophorone is very fast and thermodynamicly more favorable than the hydrogenation of C = O bond^30^. Among the investigated catalysts, the Pd/C catalyst has the highest activity and selectivity for the hydrogenation of isophorone to 3,3,5-trimethylcyclohexanone. Over this catalyst, high carbon yield of compound **2** (99.0%) was achieved after the reaction was carried out at 298 K for 1 h. According to literature^31, 32^, this result can be explained because Pd is more active and selective than Ir, Pt, Ru for the hydrogenation of C = C bond in unsaturated carbonyl compounds. The higher activity Pd for the hydrogenation of C = C bond can be explained by the stronger H_2_/metal interactions accompanied by a preferred formation of surface hydrogen atoms^33^. In the hydrogenation product over the Ir/C catalyst, small amount of 3,3,5-trimethylcyclohexanol (*i.e*. the compound **3** in Fig. 1) was also detected (see Supplementary Figs S1 and S3). This compound was produced from the simultaneous hydrogenation of C = C and C = O bonds in isophorone molecule (see Fig. 1).

### Synthesis of 3,5,5-trimethyl-2-(3,3,5-trimethylcyclohexylidene)cyclohexanone

In the second part of this work, we explored the self-aldol condensation of compound **2** under the catalysis of NaOH. From the analysis of GC-MS (Supplementary Figs S4 and S5), 3,5,5-trimethyl-2-(3,3,5-trimethyl-cyclohexylidene)cyclohexanone (*i.e*. compound **4** in Fig. 1) was identified as the major product from this reaction. No C_27_ oxygenates from the trimerization of compound **2** was detected in the product, which can be explained by the conjugate chemical structure of compound **4**. The compound **4** as obtained exists as a liquid at room temperature (see Supplementary Fig. S6). Therefore, it can be directly used for the HDO process without using any solvent.

**Figure 3:**
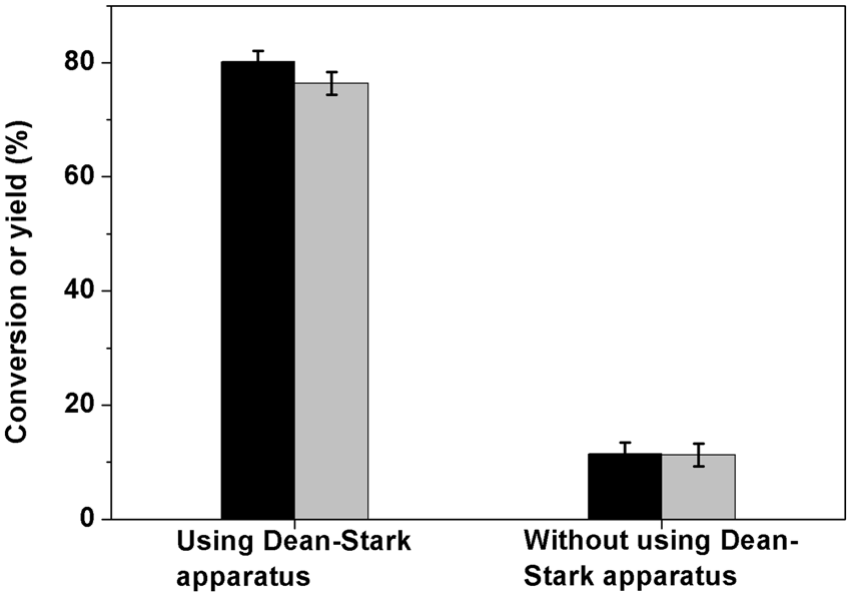
Conversion of compound 2 (black bars) and the carbon yield of compound 4 (gray bars) under the catalysis of NaOH. Reaction conditions: 443 K, 72 h; 20.0 g (0.143 mol) compound 2, 50 mmol NaOH and 20 mL *p*-xylene.

From the Fig. 3, it was noticed that the utilization of Dean-Stark apparatus is beneficial for the generation of compound **4** from the self-aldol condensation of compound **2**. This result can be comprehended from the point view of reaction equilibrium. As we know, the aldol condensation is a reversible reaction. Therefore, the removal of water from the reaction system is favorable for the generation of compound **4** and the restraining of retro-aldol condensation reaction.

**Figure 4:**
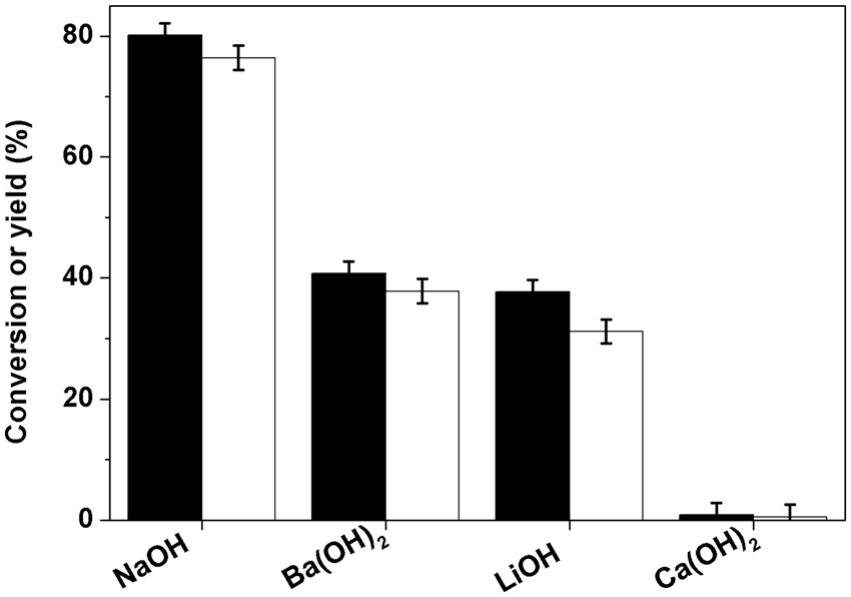
Conversion of compound 2 (black bars), the carbon yields of compound 4 (gray bars) over base catalysts. Reaction conditions: 443 K, 72 h; 20.0 g (0.143 mol) compound **2**, 50 mmol alkali hydroxide (or 25 mmol alkaline earth metal hydroxide) and 20 mL *p*-xylene.

Subsequently, we also compared the activity of series of base catalysts for the self-aldol condensation of compound **2** (see Fig. 4). Among them, NaOH exhibited the highest activity. Over this catalyst, high carbon yield of compound **4** (76.4%) was achieved after the reaction was carried out at 443 K for 72 h. The activity of base catalysts decrease in the order of NaOH > Ba(OH)_2_ > LiOH > Ca(OH)_2_ which is basically consistent with the base strength (see pK_b_ values in Supplementary Table S6) sequence of these catalysts.

### Synthesis of 1,1,3-trimethyl-5-(2,4,4-trimethylcyclohexyl)cyclohexane

**Figure 5:**
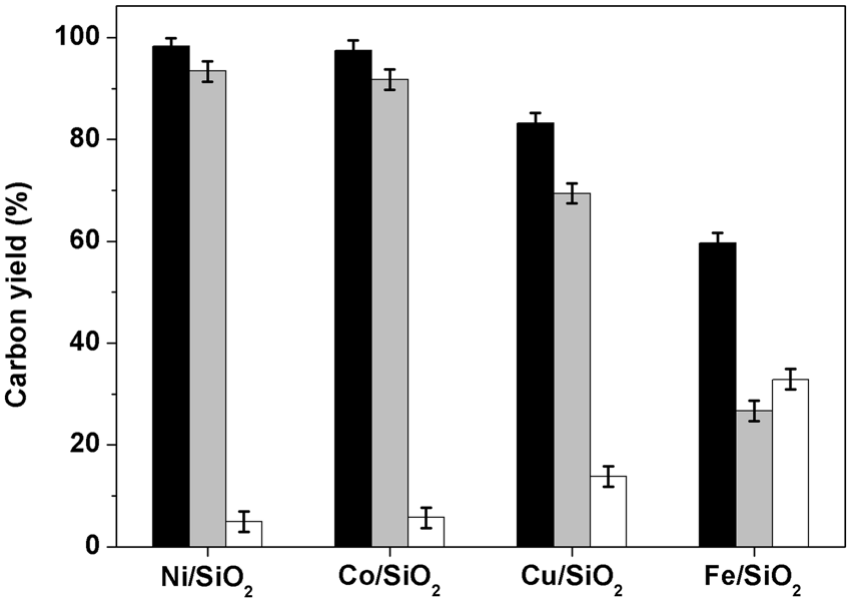
Carbon yields of compound 5 (gray bars), C_9_-C_17_ cycloalkanes (white bars) and cycloalkanes (*i.e*. the sum of compound 5 and C_9_-C_17_ cycloalkanes) (black bars), from the solvent-free HDO of compound 4 over the SiO_2_ loaded non-noble metal catalysts. Reaction conditions: 573 K, 6 MPa H_2_; 1.8 g catalyst, compound 4 flow rate: 0.04 mL min^−1^, hydrogen flow rate: 120 mL min^−1^.

**Figure 6:**
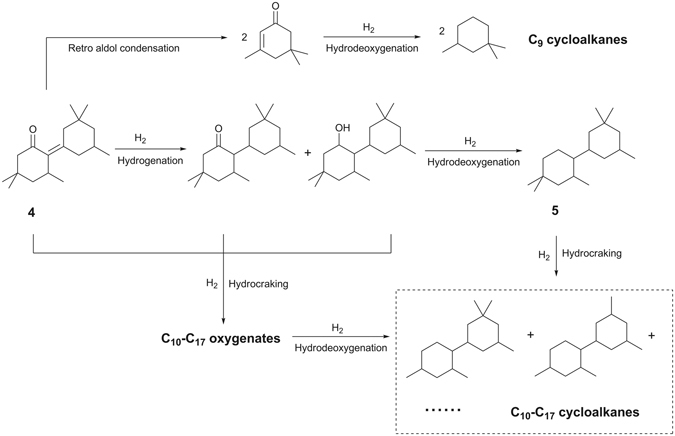
Reaction pathways for the production of different cycloalkanes from the solvent-free HDO of compound 4.

Finally, we studied the solvent-free hydrodeoxygenation (HDO) of compound **4** over a series of SiO_2_ supported non-noble metal catalysts (see Fig. 5). Among the investigated catalysts, the Ni/SiO_2_ and Co/SiO_2_ catalysts exhibited evidently higher HDO activity than those of the Cu/SiO_2_ and Fe/SiO_2_ catalysts. Over the Ni/SiO_2_ and Co/SiO_2_ catalysts, compound **4** was completed hydrodeoxygenated at 573 K, high carbon yield (93.4% and 91.8%) of 1,1,3-trimethyl-5-(2,4,4-trimethylcyclohexyl)cyclohexane (*i.e*. compound **5**) was achieved. Besides compound **5**, small amount of C_9_-C_17_ alkanes (such as 1,1,3-trimethylcyclohexane and 1,1,3-trimethyl-5-(2,4-dimethylcyclohexyl)cyclohexane) were also identified in the HDO product (see Supplementary Figs S7–S10). According to literature^34, 35^, these C_9_-C_17_ cycloalkanes may be generated by the C-C cleavage reactions (such as retro-aldol condensation, hydrocracking, *etc*.) during the HDO process. The reaction pathways for the generation of different alkanes from the HDO process were proposed in Fig. 6. According to our measurement, the cycloalkane mixture as obtained has a density of 0.858 g mL^−1^ and a freezing point of 222.2 K. As a potential application, it can be blended into conventional high density fuels for rocket propulsion. Compared with the RP-1 fuel (which is widely used as the first-stage boosters or the propellant for many rockets^14^) and other lignocellulose derived dicycloalkanes (such as dicyclohexane^23^ and dicyclopentane^19–21^) which has been reported in recent literature (see Supplementary Table S3), the compound **5** obtained in this work has higher density or lower freezing point, which is advantage in the real application.

**Figure 7:**
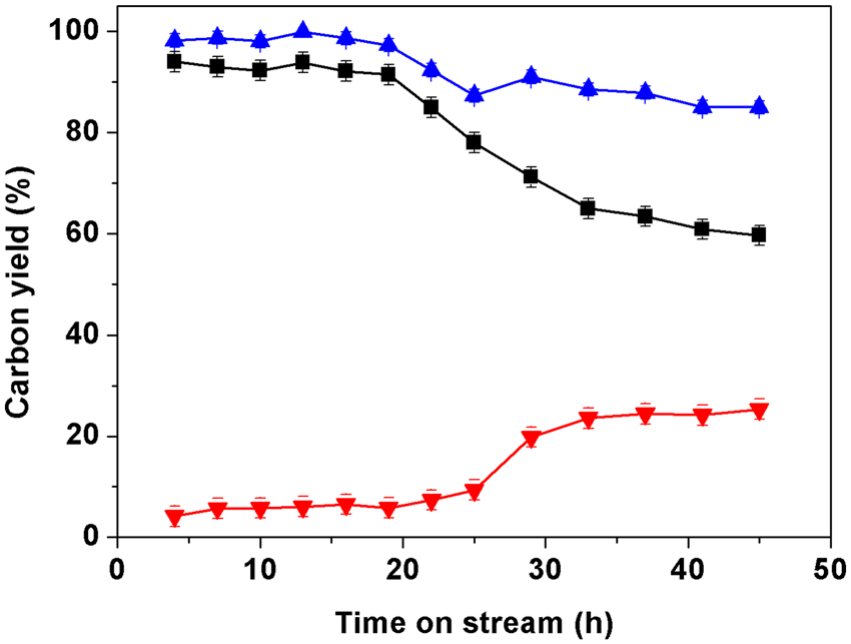
Carbon yields of compound 5 (■), C_9_-C_17_ cycloalkanes () and cycloalkanes (*i.e*. the sum of compound 5 and C_9_-C_17_ cycloalkanes) (), from the solvent-free HDO of compound 4 over the Ni/SiO_2_ catalyst as the function of reaction time. Reaction conditions: 573 K, 6.0 MPa H_2_; 1.80 g Ni/SiO_2_ catalyst, compound 4 flow rate: 0.04 mL min^−1^, hydrogen flow rate: 120 mL min^−1^.

To fulfil the need of real application, we also studied the stability of the Ni/SiO_2_ catalyst under the investigated conditions. As we can see from Fig. 7, the Ni/SiO_2_ catalyst is stable in the first 19 h. With the further increase of reaction time from 19 h to 45 h, the carbon yields of compound **5** and cycloalkanes over the Ni/SiO_2_ catalyst decreased, while the carbon yield of C_9_-C_17_ cycloalkanes slightly increased. According to the characterization of fresh and used Ni/SiO_2_ catalyst (see supplementary Table S4), this phenomenon can be explained by the aggregation of Ni particles during the HDO test.

## Conclusions

Herein, we reported a new route for the synthesis of a renewable high density dicycloalkane, 1,1,3-trimethyl-5-(2,4,4-trimethylcyclohexyl)cyclohexane with isophorone. In the first step, isophorone was converted to 3,3,5-trimethylcyclohexanone by selective hydrogenation. Over the Pd/C catalyst, 99.0% carbon yield of 3,3,5-trimethylcyclohexanone was obtained under mild conditions. In the second step, 3,5,5-trimethyl-2-(3,3,5-trimethylcyclohexylidene)-cyclohexanone was synthesized by the self-aldol condensation of 3,3,5-trimethylcyclohexanone under the catalysis of NaOH. The utilization of Dean-Stark apparatus is favorable for this reaction. In the third step, the 3,5,5-trimethyl-2-(3,3,5-trimethylcyclohexylidene)cyclohexanone as further hydrodeoxygenated under solvent-free conditions. High carbon yields of 1,1,3-trimethyl-5-(2,4,4-trimethylcyclohexyl)cyclohexane (93.4%) was achieved over the Ni/SiO_2_ catalyst at 573 K. The polycycloalkanes mixture as obtained has a density of 0.858 g mL^−1^ and a freezing point of 222.2 K. As a potential application, it can be blended into conventional cycloalkane fuels (such as RP-1) for rockets propulsion.

## Methods

### Preparation of catalysts

The Pd/C, Ir/C, Pt/C, Ru/C, LiOH, NaOH, Ca(OH)_2_ and Ba(OH)_2_ catalysts are commercial available. The Ni/SiO_2_, Co/SiO_2_, Cu/SiO_2_ and Fe/SiO_2_ catalysts used in the hydrodeoxygenation (HDO) process were prepared by the method described in supporting information.

### Activity test

The hydrogenation of 3,3,5-trimethylcyclohexanone was conducted with a stainless steel batch reactor. The self-aldol condensation of 3,3,5-trimethylcyclohexanone was carried out in a flask which was attached to the Dean-Stark apparatus to remove the water generated during the reaction. The solvent-free HDO of 3,5,5-trimethyl-2-(3,3,5-trimethylcyclohexylidene)cyclohexanone was conducted at 573 K using a fixed-bed continuous flow reactor. The detail information for the activity tests was described in supporting information.

## Electronic supplementary material

Supplementary Information

## References

[1] Huber GW, Iborra S, Corma A (2006). Synthesis of transportation fuels from biomass: Chemistry, catalysts, and engineering. Chem. Rev..

[2] Matson TD, Barta K, Iretskii AV, Ford PC (2011). One-pot catalytic conversion of cellulose and of woody biomass solids to liquid fuels. J. Am. Chem. Soc..

[3] Xia Q (2016). Direct hydrodeoxygenation of raw woody biomass into liquid alkanes. Nat. Commun..

[4] Corma A, Iborra S, Velty A (2007). Chemical routes for the transformation of biomass into chemicals. Chem. Rev..

[5] Wang X, Rinaldi R (2012). Exploiting H-transfer reactions with RANEY^®^ Ni for upgrade of phenolic and aromatic biorefinery feeds under unusual, low-severity conditions. Energy Environ. Sci..

[6] Wang YL (2013). Chemical synthesis of lactic acid from cellulose catalysed by lead(II) ions in water. Nat. Commun..

[7] Liu F (2014). Palladium/carbon dioxide cooperative catalysis for the production of diketone derivatives from carbohydrates. ChemSusChem.

[8] Van den Bosch S (2015). Reductive lignocellulose fractionation into soluble lignin-derived phenolic monomers and dimers and processable carbohydrate pulps. Energy Environ. Sci..

[9] Prasomsri T, Shetty M, Murugappan K, Roman-Leshkov Y (2014). Insights into the catalytic activity and surface modification of MoO_3_ during the hydrodeoxygenation of lignin-derived model compounds into aromatic hydrocarbons under low hydrogen pressures. Energy Environ. Sci..

[10] Chung HS, Chen CSH, Kremer RA, Boulton JR, Burdette GW (1999). Recent developments in high-energy density liquid hydrocarbon fuels. Energy Fuels.

[11] Zou J-J, Zhang X, Kong J, Wang L (2008). Hydrogenation of Dicyclopentadiene over amorphous nickel alloy catalyst SRNA-4. Fuel.

[12] Zou J-J, Xiong Z, Wang L, Zhang X, Mi Z (2007). Preparation of Pd-B/γ-Al_2_O_3_ amorphous catalyst for the hydrogenation of tricyclopentadiene. J. Mol. Catal. A: Chem..

[13] Zou J-J (2007). Kinetics of Tricyclopentadiene Hydrogenation over Pd-B/γ-Al_2_O_3_ Amorphous Catalyst. Ind. Eng. Chem. Res..

[14] Striebich RC, Lawrence J (2003). Thermal decomposition of high-energy density materials at high pressure and temperature. J. Anal. Appl. Pyrol..

[15] Harvey BG, Wright ME, Quintana RL (2010). High-density renewable fuels based on the selective dimerization of pinenes. Energy Fuels.

[16] Meylemans HA, Quintana RL, Goldsmith BR, Harvey BG (2011). Solvent-free conversion of linalool to methylcyclopentadiene dimers: a route to renewable high-density fuels. ChemSusChem.

[17] Zou JJ, Chang N, Zhang XW, Wang L (2012). Isomerization and dimerization of pinene using Al-incorporated MCM-41 mesoporous materials. ChemCatChem.

[18] Nie GK, Zou JJ, Feng R, Zhang XW, Wang L (2014). HPW/MCM-41 catalyzed isomerization and dimerization of pure pinene and crude turpentine. Catal. Today.

[19] Yang Y (2013). Conversion of furfural into cyclopentanone over Ni-Cu bimetallic catalysts. Green Chem..

[20] Deng Q (2015). Highly selective self-condensation of cyclic ketones using MOF-encapsulating phosphotungstic acid for renewable high-density fuel. Green Chem..

[21] Yang J (2014). Synthesis of renewable high-density fuels using cyclopentanone derived from lignocellulose. Chem. Commun..

[22] Deng Q (2016). Efficient synthesis of high-density aviation biofuel via solvent-free aldol condensation of cyclic ketones and furanic aldehydes. Fuel Process. Technol..

[23] Zhao C, Camaioni DM, Lercher JA (2012). Selective catalytic hydroalkylation and deoxygenation of substituted phenols to bicycloalkanes. J. Catal..

[24] Nie G (2017). Lignin-derived multi-cyclic high density biofuel by alkylation and hydrogenated intramolecular cyclization. Chem. Eng. Sci..

[25] Deng Q (2015). Highly controllable and selective hydroxyalkylation/alkylation of 2-methylfuran with cyclohexanone for synthesis of high-density biofuel. Chem. Eng. Sci..

[26] Zhang X (2017). Hydrophobic mesoporous acidic resin for hydroxyalkylation/alkylation of 2-methylfuran and ketone to high-density biofuel. AIChE J..

[27] Kelkar CP, Schutz AA (1998). Efficient hydrotalcite-based catalyst for acetone condensation to α-isophorone—scale up aspects and process development. Appl. Clay Sci..

[28] Mahata N, Cunha AF, Órfão JJM, Figueiredo JL (2012). Highly selective hydrogenation of C = C double bond in unsaturated carbonyl compounds over NiC catalyst. Chem. Eng. J..

[29] Sato T, Rode CV, Sato O, Shirai M (2004). Hydrogenation of isophorone with noble metal catalysts in supercritical carbon dioxide. Appl. Catal. B: Environ.

[30] Gallezot P, Richard D (1998). Selective hydrogenation of α,β-unsaturated aldehydes. Catal. Rev.-Sci. Eng..

[31] Delbecq F, Sautet P (1995). Competitive C = C and C = O adsorption of α,β-unsaturated aldehydes on Pt and Pd surfaces in relation with the selectivity of hydrogenation reactions: a theoretical approach. J. Catal..

[32] Ganji S, Mutyala S, Neeli CKP, Rao KSR, Burri DR (2013). Selective hydrogenation of the C = C bond of α,β-unsaturated carbonyl compounds over PdNPs-SBA-15 in a water medium. RSC Adv..

[33] Obenaus U (2016). Relationships between the Hydrogenation and Dehydrogenation Properties of Rh-, Ir-, Pd-, and Pt-Containing Zeolites Y Studied by *In Situ* MAS NMR Spectroscopy and Conventional Heterogeneous Catalysis. J Phys Chem C.

[34] Li N, Huber GW (2010). Aqueous-phase hydrodeoxygenation of sorbitol with Pt/SiO_2_-Al_2_O_3_: Identification of reaction intermediates. J. Catal..

[35] Li G (2014). Synthesis of renewable diesel range alkanes by hydrodeoxygenation of furans over Ni/Hβ under mild conditions. Green Chem..

