# Supplementary material for: Synthesis of renewable high-density fuel with isophorone
Source: Sci Rep. 2017 Jul 21;7:6111. doi: 10.1038/s41598-017-06556-7 (PMC5522473; doi:10.1038/s41598-017-06556-7)
Supplement: Supplementary file 1 — Supplementary Information [file 41598_2017_6556_MOESM1_ESM.pdf]

Supporting information

Synthesis of renewable high-density fuel with isophorone

Wei Wang,[1,2,§] Yanting Liu,[2,§] Ning Li,[2,3]* Guangyi Li,[2] Wentao Wang,[2] Aiqin Wang,[2,3] Xiaodong Wang,[2] and Tao Zhang [2,3]*

[1] Shaanxi Key Laboratory of Catalysis, School of Chemistry and Environment Science, Shaanxi University of Technology, No. 1 Dongyihuan Road, Hanzhong 723001, China

[2] State Key Laboratory of Catalysis, Dalian Institute of Chemical Physics, Chinese Academy of Sciences, No. 457 Zhongshan Road, Dalian 116023, China

[3] *iChEM* (Collaborative Innovation Centre of Chemistry for Energy Materials), Dalian Institute of Chemical Physics, Chinese Academy of Sciences, No. 457 Zhongshan Road, Dalian 116023, China

[§] These authors contributed equally to this work.

*Corresponding author: E-mail: taozhang@dicp.ac.cn (T. Zhang) or lining@dicp.ac.cn (N. Li)

Catalyst preparation. Analytical-grade isophorone (purity: 97%), commercial available Pd/C, Ir/C, Pt/C, Ru/C used in the selective hydrogenation tests were supplied by Shanghai Aladdin Bio-Chem Technology Co., LTD. To facilitate comparison, the metal contents in the activated carbon loaded noble metal catalysts were fixed at 5% by weight (denoted as 5wt.%). The LiOH, NaOH, $Ca(OH)_2$ and $Ba(OH)_2$ catalysts (analytical-grade, purity > 95%) used in the self-aldol condensation of 3,3,5-trimethylcyclohexanone were purchased from Tianjin Kermel Chemical Reagent Co., Ltd. The Ni/SiO_2, Co/SiO_2, Cu/SiO_2 and Fe/SiO_2 catalysts used in the hydrodeoxygenation (HDO) process were prepared by the incipient wetness impregnation of commercial available SiO_2 support (Qingdao Ocean Chemical Ltd., BET surface area 463 m^2 g^{-1}, purity: 99%) with the aqueous solutions of analytical-grade $Ni(NO_3)_3 \cdot 6H_2O$, $Co(NO_3)_3 \cdot 6H_2O$, $Cu(NO_3)_2 \cdot 6H_2O$ and $Fe(NO_3)_3 \cdot 9H_2O$, (purchased from Sinopharm Chemical Reagent Co., LTD, purity > 98%), respectively. To facilitate comparison, the theoretical metal contents in these catalysts were fixed at 5% by weight (denoted as 5wt.%). After being dried at 353 K for 4 h, the catalysts were calcined at 773 K for 4 h.

Activity tests. *Synthesis of 3,3,5-trimethylcyclohexanone.* The synthesis of 3,3,5-trimethylcyclohexanone (*i.e.* compound **2** in Fig. 1) by the selective hydrogenation of isophorone was conducted with a stainless steel batch reactor. For each test, 1.16 g (8.4 mmol) isophorone and 0.05 g activated carbon loaded noble metal catalyst (directly used without any pretreatment) were utilized. Before the test, the stainless steel batch reactor was purged by argon for three times. Subsequently,

hydrogen was introduced into the batch reactor until the system pressure reached 2.0 MPa. The mixture of catalyst and reactant was stirred at 298 K for 1 h. After the releasing of unreacted hydrogen, the liquid products were collected from the stainless steel batch reactor, filtrated to remove the solid catalyst and analyzed by an Agilent 7890A gas chromatograph (GC) which was equipped with a HP-INNOWAX capillary column (30 m, 0.25 mm ID, 0.5 mm film) and a flame ionization detector (FID). The oven temperature of the GC was held at 313 K for 2 min, increased to 553 K at a rate of 15 K min^{-1}, and stayed at that temperature for 5 min. Helium was used as the carrier gas at a flow rate of 1.5 mL min^{-1}.

Conversion of isophorone (%) = [1 - (Mol of unreacted isophorone in the product)/(Mol of isophorone in the feedstock)] × 100%

Carbon yield of hydrogenation product (%) = (Carbon in the certain hydrogenation product)/(Carbon in the isophorone feedstock) × 100%

Synthesis of 3,5,5-trimethyl-2-(3,3,5-trimethylcyclohexylidene)cyclohexanone. The self-aldol condensation of compound **2** to 3,5,5-trimethyl-2-(3,3,5-trimethylcyclohexylidene)cyclohexanone (*i.e.* compound **4** in Fig. 1) was carried out in a flask which was attached to the Dean-Stark apparatus to remove the water generated during the reaction (see Fig. S11). Typically, 20.0 g (0.143 mol) compound **2**, 50 mmol alkali hydroxide (or 25 mmol alkaline earth metal hydroxide) and 20 mL *p*-xylene were vigorously stirred at 443 K for 72 h. After the reaction, the liquid product was neutralized with 50wt.% H_3PO_4 solution, diluted and analyzed by the Agilent 7890A GC which was used for the analysis of hydrogenation product. For

comparison, we also investigated the self-aldol condensation of compound **2** without using the Dean-Stark apparatus under the same reaction conditions.

Conversion of compound **2** (%) = [1- (Mol of unreacted compound **2** in the product)/(Mol of compound **2** in the feedstock)] × 100%

Carbon yield of compound **4** (%) = (Carbon in the compound **4** obtained)/(Carbon in the compound **2** feedstock) × 100%

*Hydrodeoxygenation (HDO)***.** The solvent-free HDO of 3,5,5-trimethyl-2-(3,3,5-trimethylcyclohexylidene)cyclohexanone was conducted at 573 K using a fixed-bed continuous flow reactor. For each test, 1.8 g M/SiO_2 (M = Ni, Co, Cu, Fe) catalyst was used. Before the test, the catalyst was *in-situ* reduced by H_2 flow (120 mL min^{-1}) at 733 K for 2 h. After the reactor temperature decreased to 573 K and stabilized at that value for 0.5 h. The 3,5,5-trimethyl-2-(3,3,5-trimethylcyclohexylidene)cyclohexanone (purified from the self-aldol condensation product by vacuum distillation) was fed into the reactor (at a rate of 0.04 mL min^{-1}) along with H_2 (at a rate of 120 mL min^{-1}). After passing through a gas-liquid separator and a back pressure regulator (which was used to maintain the system pressure at 6 MPa), the gas phase products were analyzed online by an Agilent 6890N GC. The CO_2 in the gaseous product was separated by a RESTEK HS-DB 100/120 packed column (30 feet, 1/8 inch outer diameter, 2.0 mm inner diameter) using helium as the carrier gas (flow rate: 24 mL min^{-1}) and analyzed by a Thermal Conductivity Detector (TCD). The TCD and the injection port of the GC were held at 523 K and 393 K, respectively. The alkanes in the gaseous product

were analyzed by a FID which was connected with a RESTEK Rt-Q-BOND capillary column (30 m, 0.32 mm ID, 10 μm film) using helium as the carrier gas (flow rate of 1 mL min^{-1}). The oven temperature of the GC was held at 323 K for 2 min, then increased to 473 K at a rate of 15 K min^{-1} and kept at that temperature for 10 min. The liquid phase products were collected periodically from the bottom of separator, diluted and analyzed by the Agilent 7890A GC which was used for the analysis of condensation products.

Conversion of compound **4** (%) = [1- (Mol of unreacted compound **4** in the product)/(Mol of compound **4** in the feedstock)] × 100%

Carbon yield of certain cycloalkanes (%) = (Carbon in the certain cycloalkane product)/(Carbon in the compound **4** feedstock) × 100%

Characterization of catalysts.

N_2-physisorption. The specific Brunauer-Emmet-Teller (BET) surface areas of the catalysts were measured by nitrogen physisorption at 77 K using an ASAP 2010 apparatus. Before each measurement, the sample was evacuated at 573 K for 3 h.

H_2-chemisorption. The metal dispersions and metallic surface areas of the HDO catalysts were characterized by H_2 chemisorption which was carried out with a Micromeritics Autochem II 2920 automated chemisorption analyzer. Before each test, the sample was reduced in 10vol.% H_2/Ar flow at 773 K for 2 h. Subsequently, the sample was purged with Ar flow at 783 K for 0.5 h and cooled down in Ar flow to 323 K. After the stabilization of baseline, the H_2 adsorption was carried out by the pulse adsorption of 10vol.% H_2/Ar at 323 K.

Aldol condensation:

Initially, we want to synthesize the C_{18} oxygenate by the self-aldol condensation of isophorone. However, the reactivity of this compound is very low. No self-aldol condensation product (or C_{18} oxygenate) was obtained under the investigated conditions (using Dean-Stark apparatus), which can be rationalized by the conjugate structure of isophorone. On the basis of this result, we think the hydrogenation (or saturation of C=C bond) of isophorone is necessary to increase the reactivity of this compound which is favorable for the production of C_{18} oxygenate by self-aldol condensation.

To figure out whether the self-aldol condensation of compound **2** follows a homogeneously or heterogeneously-catalyzed pathway, we did some experiments using Dean-Stark apparatus. Firstly, we stirred the mixtures of compound **2** and alkali hydroxide (or alkaline earth metal hydroxide) under the investigated conditions for 10 min. It was found that the alkali hydroxide and alkaline earth metal hydroxide are insoluble in the mixture of compound **2** and *p*-xylene. The reaction systems automatically separated into two phases after we stopped the stirring. Using the organic phases which were quickly separated from the alkali hydroxide (or alkaline earth metal hydroxide) by decantation, we conducted the self-aldol condensation under the same reaction conditions (443 K for 72 h). From the analysis of products, no compound **4** from the self-aldol condensation of compound **2** was identified in the products. Based on the result, we believe the self-aldol condensation of compound **2** follow a heterogeneously-catalyzed pathway. In real application, the Dean-Stark

apparatus can be replaced by a fix-bed continuous flow reactor in which the water generated during the reaction can be removed by carrier gas. Due to their low densities and low water-solubility, the compound **4** as obtained and the unreacted compound **2** can be spontaneously separated from the water in an oil-water separator. After being separated by distillation, the compound **4** was used for the subsequent HDO process. The unreacted compound **2** can be fed back into the reactor to increase the carbon yield of compound **4**.

Table S1. Hemicellulose contents in various terrestrial biomasses.

Biomass	Hemicellulose content (wt.%)[1,2]
Corn grain	6
Corn stover	23
Switchgrass	31-35
Sugarcane	15
Birch	19.3
Pine	19-22
Poplar	20.4
Basswood	28.9
Ashtree	22.7
Beech	20.7
Xylosma	21.1

According to the Table S1, we can see that corn stover, switchgrass and some woody biomasses have relatively higher hemicellulose contents, which makes them potential feedstocks in future application of current route.

Table S2. The pK_b values of the base catalysts used in the self-aldol condensation of compound 2.

Catalyst	pK_b
LiOH	0.2
NaOH	-0.48
$Ca(OH)_2$	2.43
	1.4
$Ba(OH)_2$	-[a]
	0.64

a: The pK_{b2} of $Ba(OH)_2$ is not available.

Table S3. The typical components, densities and freezing points of RP-1 fuel, RG-1 fuel, bi(cyclohexane), bi(cyclopentane) and the compound 5 obtained in this work.

	RP-1[3]	RG-1[3]	Bi(cyclohexane)[4]	Bi(cyclopentane)[5]	Compound **5** obtained in this work
Typical components	Mostly paraffins and cycloparaffins	Mostly isoparaffins and cycloparaffins	Bi(cyclohexane)	Bi(cyclopentane)	1,1,3-trimethyl-5-(2,4,4-trimethylcyclohexyl)cyclohexane
Density (g mL^{-1})	0.81	0.84	0.88	0.866	0.858
Freezing point (K)	224.1	226.3	277	237.7	222.2

Table S4. Actual Ni contents, metal dispersions and metallic surface areas of the fresh and used Ni/SiO_2 catalysts.

Catalyst	Actual Ni content (%) [a]	Metal dispersion (%) [b]	Metallic surface areas (m^2 g^{-1}) [b]
Fresh Ni/SiO_2	4.832	4.27	27.56
Used Ni/SiO_2	4.831	1.60	10.31

[a] Measured by Thermo IRIS Intrepid II inductively coupled plasma (Perkin-Elmer Optima 7300DV). [b] Calculated from the results of H_2 chemisorption.

To figure out the reason for the activity change during the long-term test, we characterized the fresh and used Ni/SiO_2 catalysts. According to the result of ICP analysis (see Table S4), the actual Ni content in the used Ni/SiO_2 catalyst (4.831wt.%) is the same as the one in fresh Ni/SiO_2 catalyst (4.832wt.%), which means that the leaching of Ni during the HDO test can be ignored. Based on the H_2-chemisorption results, the metal dispersion and metallic surface area on the surface of the Ni/SiO_2 catalyst evidently decreased after being used for 45 h. This result means that the Ni particles on the surface of the Ni/SiO_2 catalyst aggregated during the long-term test, which may be one reason for the activity change of the Ni/SiO_2 catalyst.

Table S5. Metal dispersions, specific BET surface areas (S_{BET}), pore volumes, average pore sizes of the HDO catalysts.

Catalyst	Metal dispersion (%) [a]	S_{BET} ($m^2\ g^{-1}$) [b]	Pore volume ($cm^3\ g^{-1}$) [b]	Average pore size(nm) [b]
Fe/SiO_2	5.75	522	0.80	3.27
Co/SiO_2	3.83	590	0.90	3.29
Ni/SiO_2	4.27	480	0.71	3.29
Cu/SiO_2	3.01	470	0.70	3.30

[a] Measured by the H_2-chemisorption. [b] Measured by N_2-physisorption.

From Table S5, we can see that there is no clear relationship between the activities (Ni/SiO_2 > Co/SiO_2 > Cu/SiO_2 > Fe/SiO_2) and the metal dispersions, specific BET surface areas, pore volumes or average pore sizes of these catalysts.

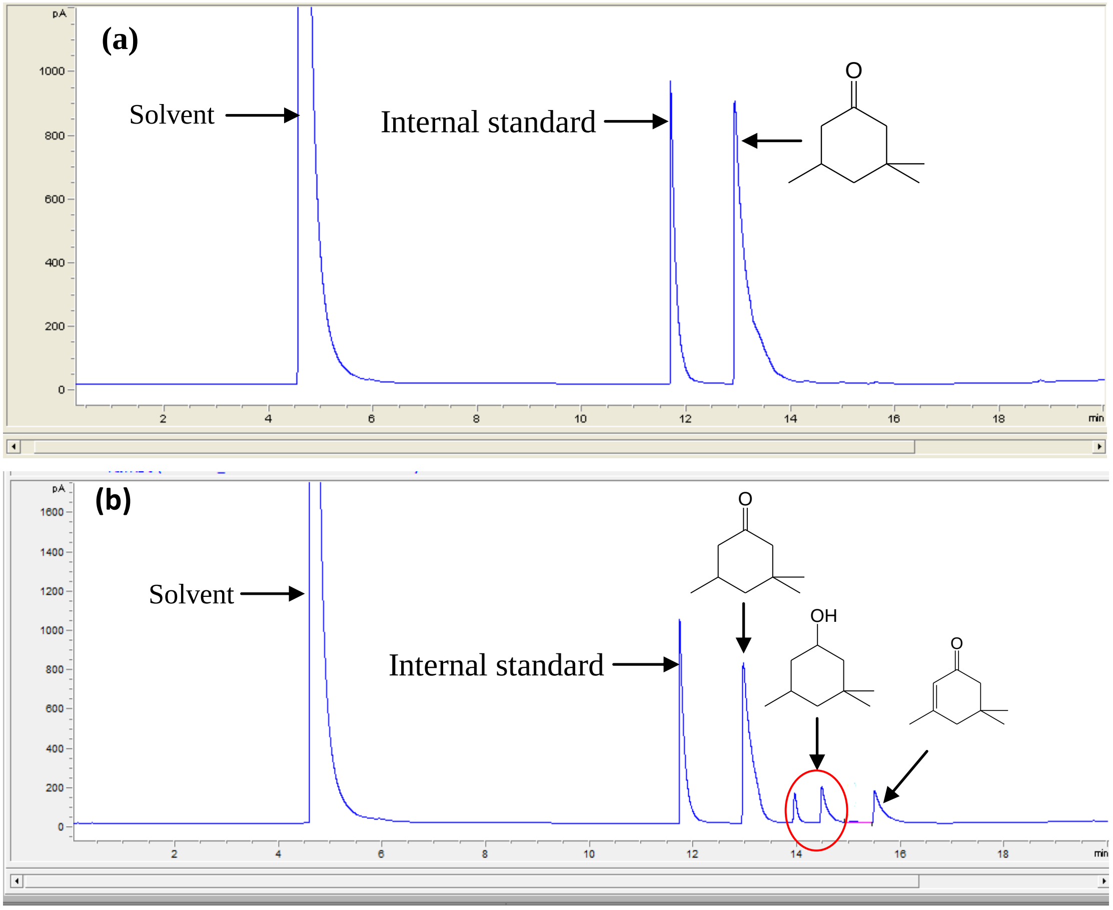

Figure S1 | GC chromatograms of the hydrogenation products of isophorone over the Pd/C (a) and Ir/C (b) catalysts. Reaction conditions: 298 K, 1 h; 1.16 g (8.4 mmol) isophorone, 0.05 g catalyst, 2.0 MPa H_2.

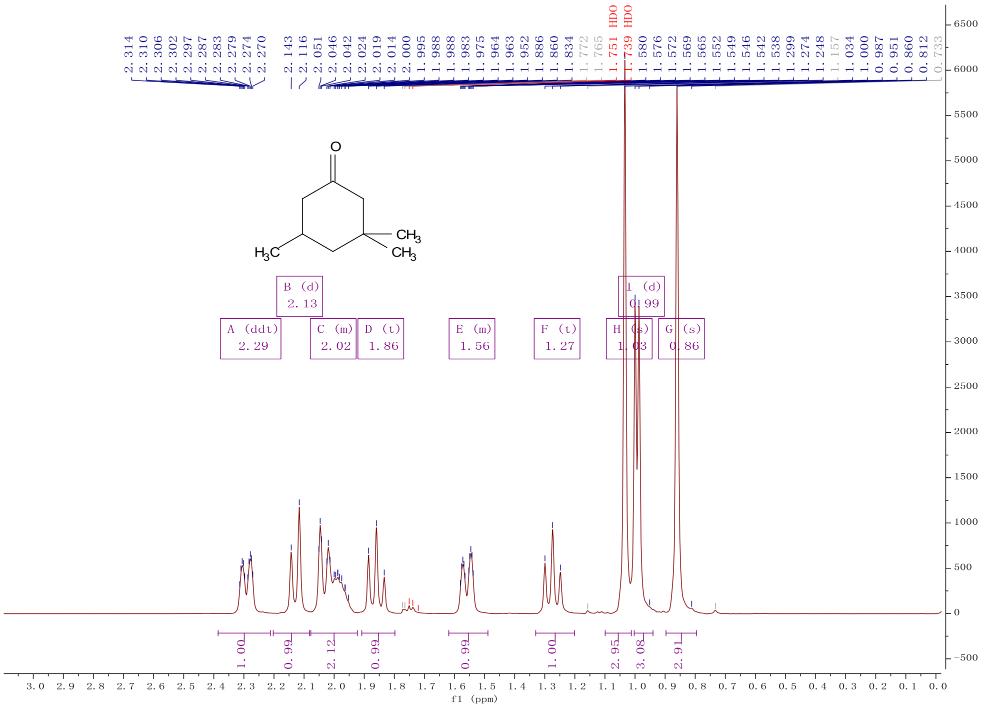

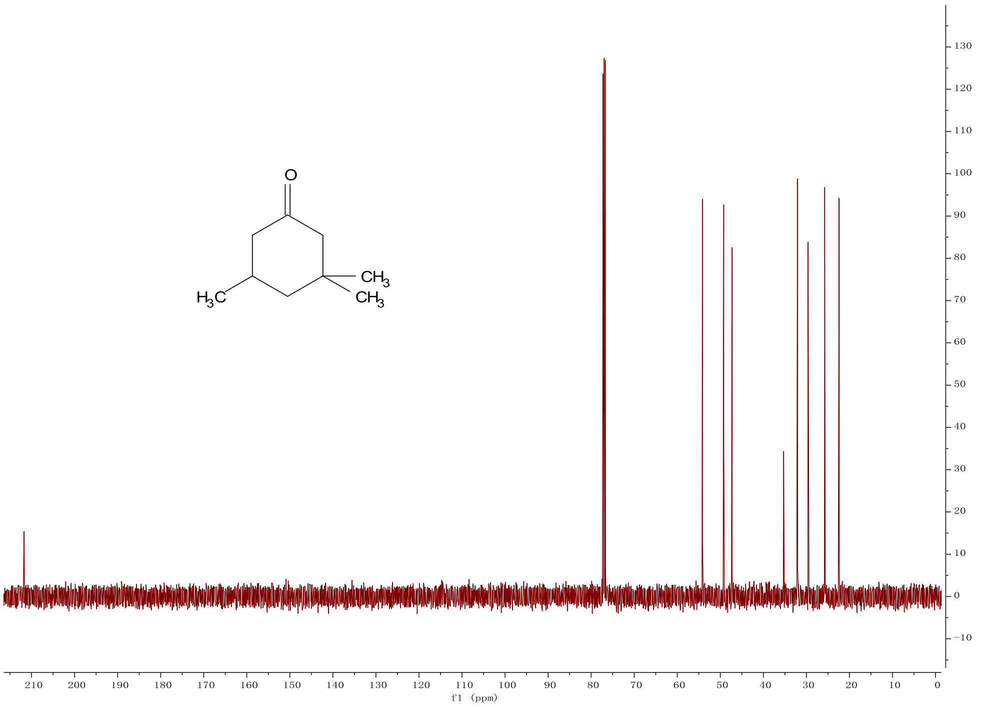

Figure S2 | ^{1}H and ^{13}C NMR spectra of the compound 3 obtained from the hydrogenation of isophorone. ^{1}H NMR (500 MHz, $CDCl_3$) δ 2.29 (d, J = 13.4 Hz, 1H, position 2), 2.13 (d, J = 13.4 Hz, 1H, position 6), 2.03 (d, J = 13.4 Hz, 1H, position 6), 2.01 – 1.93 (m, 1H, position 5), 1.86 (t, J = 13.0 Hz, 1H, position 4), 1.56 (d, J = 13.3 Hz, 1H, position 2), 1.27 (t, J = 12.8 Hz, 1H, position 4), 1.03 (s, 3H, –C–$(CH_3)_2$), 0.99 (d, J = 6.4 Hz, 3H, –CH–CH_3), 0.86 (s, 3H, –C–$(CH_3)_2$). ^{13}C NMR (126 MHz, $CDCl_3$) δ 211.93 (C1), 54.33 (C2), 49.39 (C6), 47.48 (C3), 35.47 (C5), 32.25 (C4), 29.81 (C7), 25.94 (C8), 22.59 (C9).

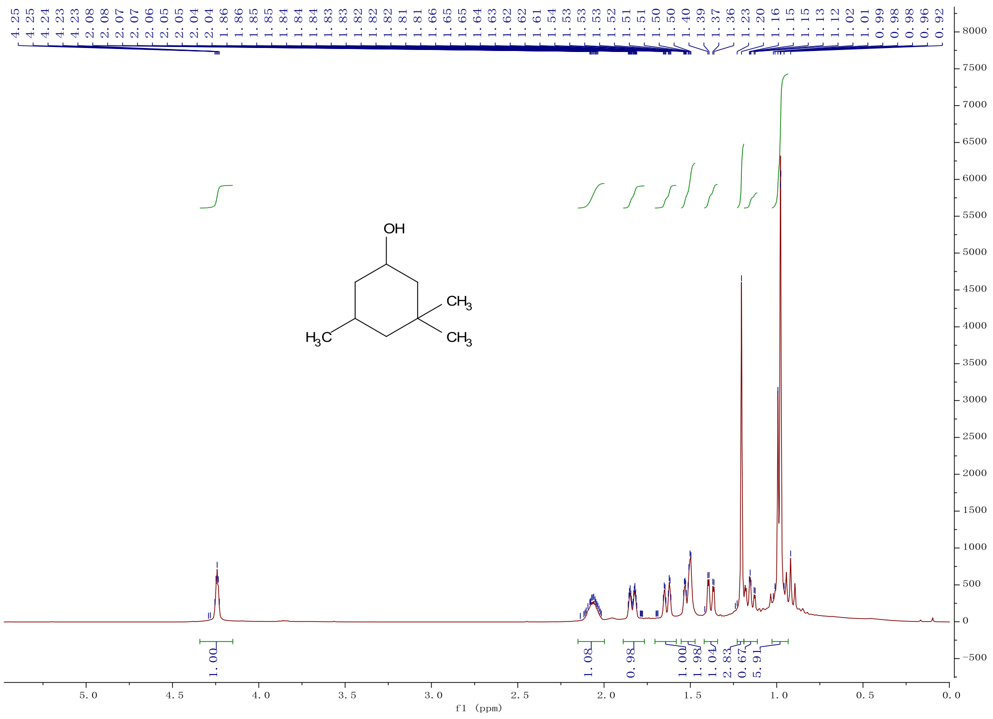

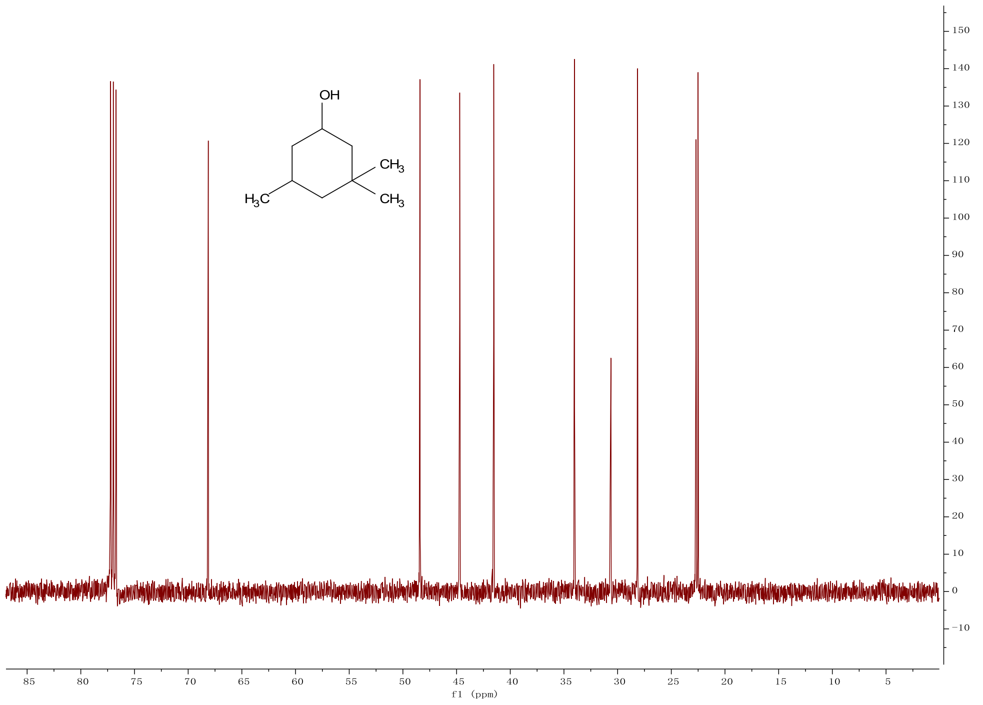

Figure S3 | ^{1}H and ^{13}C NMR spectra of the compound 3 obtained from obtained from the hydrogenation of isophorone over the Ir/C catalyst. Reaction conditions: 298 K, 10 h; 1.16 g (8.4 mmol) isophorone, 0.05 g Ir/C catalyst, 2.0 MPa H_2.

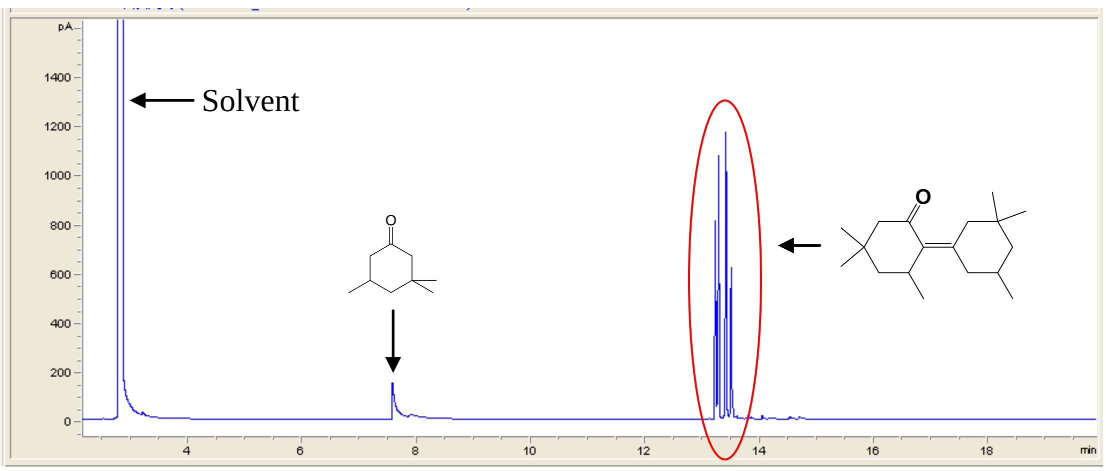

Figure S4 | GC chromatogram of the products from the self-aldol condensation of compound 2 under the catalysis of NaOH. Reaction conditions: 443 K, 72 h; 20.0 g (0.143 mol) compound **2**, 50 mmol NaOH and 20 mL *p*-xylene.

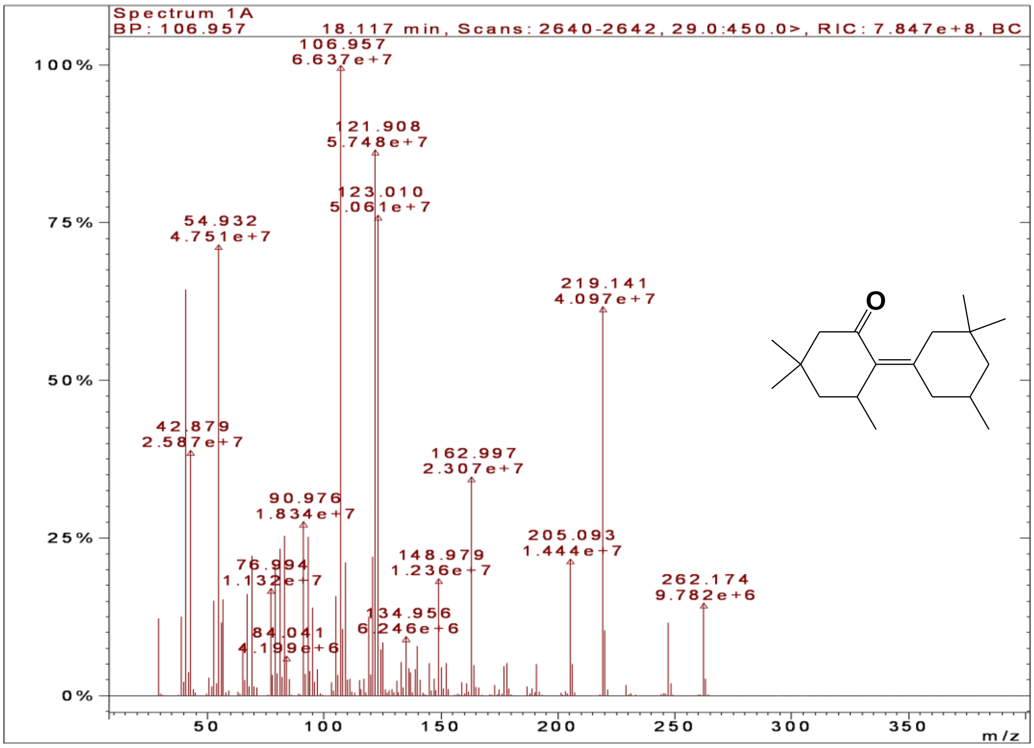

Figure S5 | Mass spectrogram of the compound 4 from the self-aldol condensation of the compound 2.

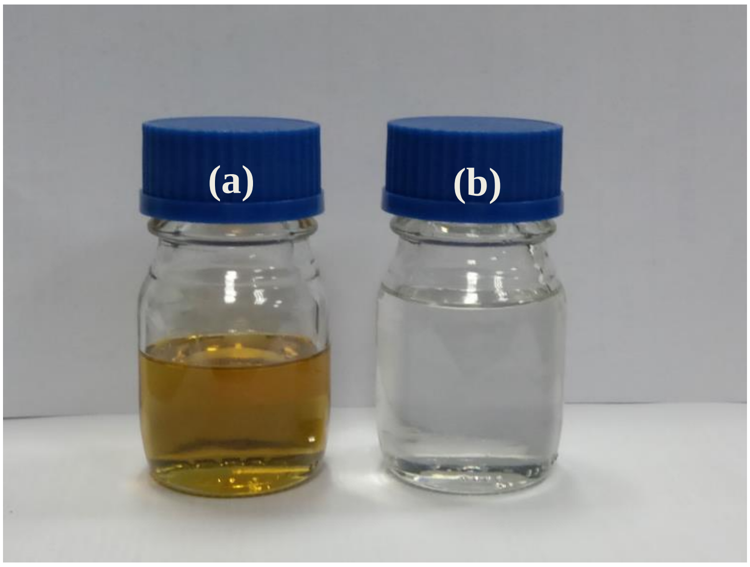

Figure S6 | Photo of the compound 4 from the self-aldol condensation of the compound 2 (a) and the HDO product of compound 4 over the Ni/SiO_2 catalyst (b).

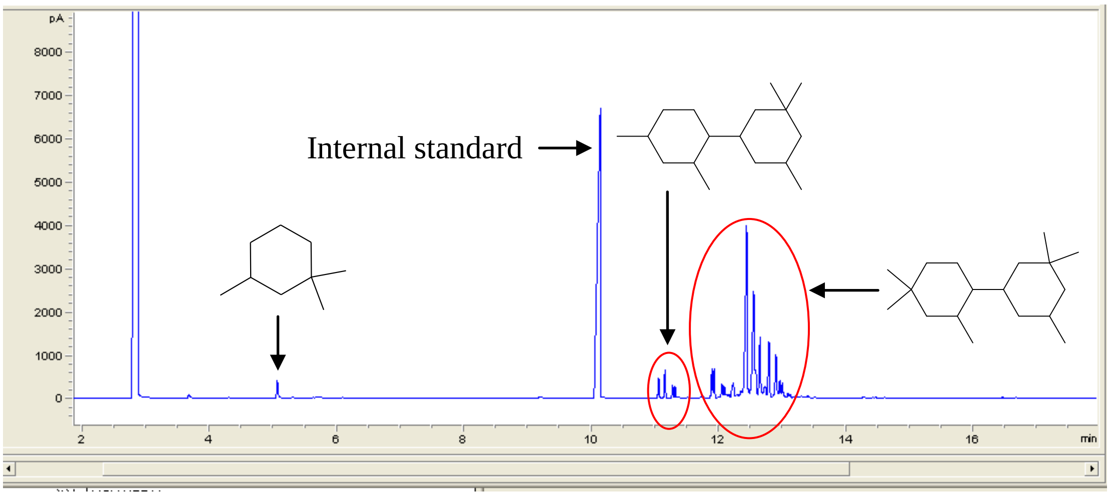

Figure S7 | GC chromatogram of the products from the solvent-free HDO of compound 4 over the Ni/SiO$_2$ catalyst. Reaction conditions: 573 K, 6.0 MPa H$_2$; 1.80 g Ni/SiO$_2$ catalyst, compound **4** flow rate: 0.04 mL min^{-1}, hydrogen flow rate: 120 mL min^{-1}.

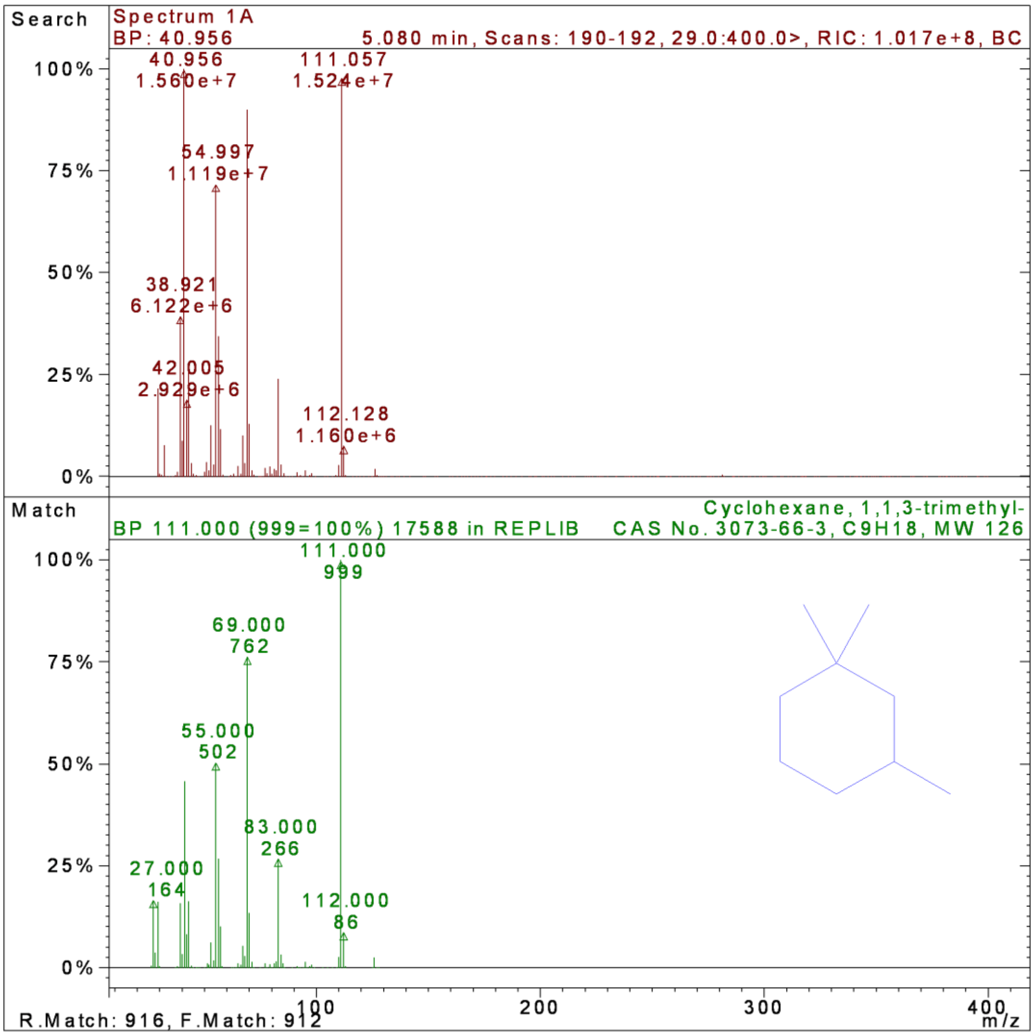

Figure S8 | Mass spectrogram of the 1,1,3-trimethylcyclohexane from the solvent-free HDO of compound 4.

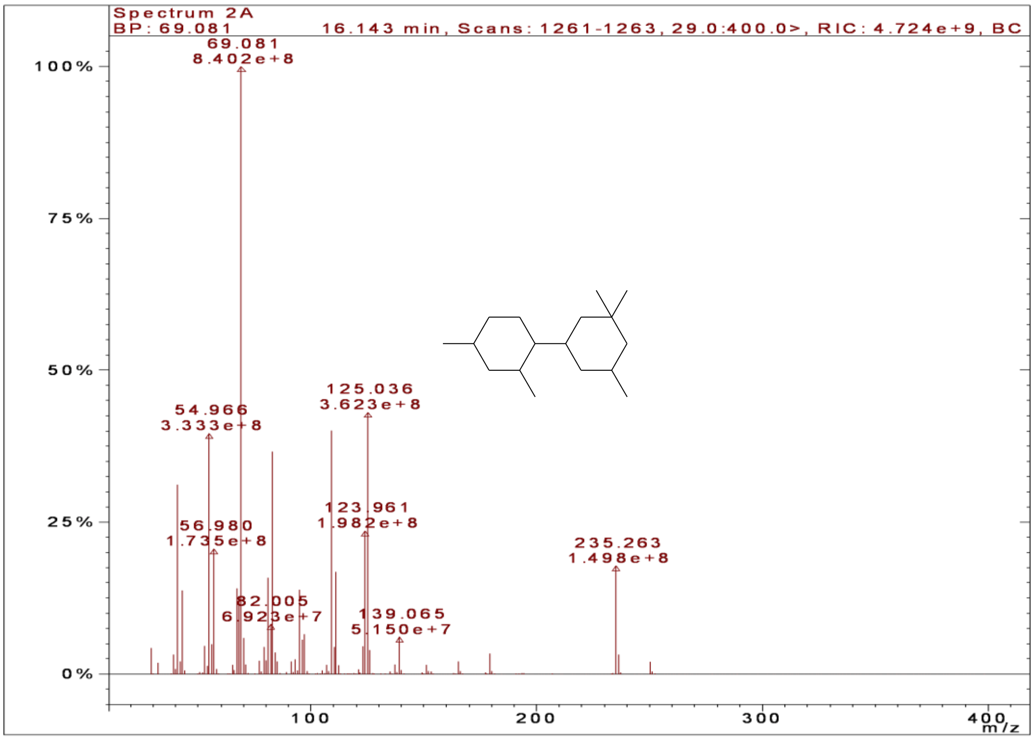

Figure S9 | Mass spectrogram of 1,1,3-trimethyl-5-(2,4-dimethyl-cyclohexyl)cyclohexane from the solvent-free HDO of compound 4.

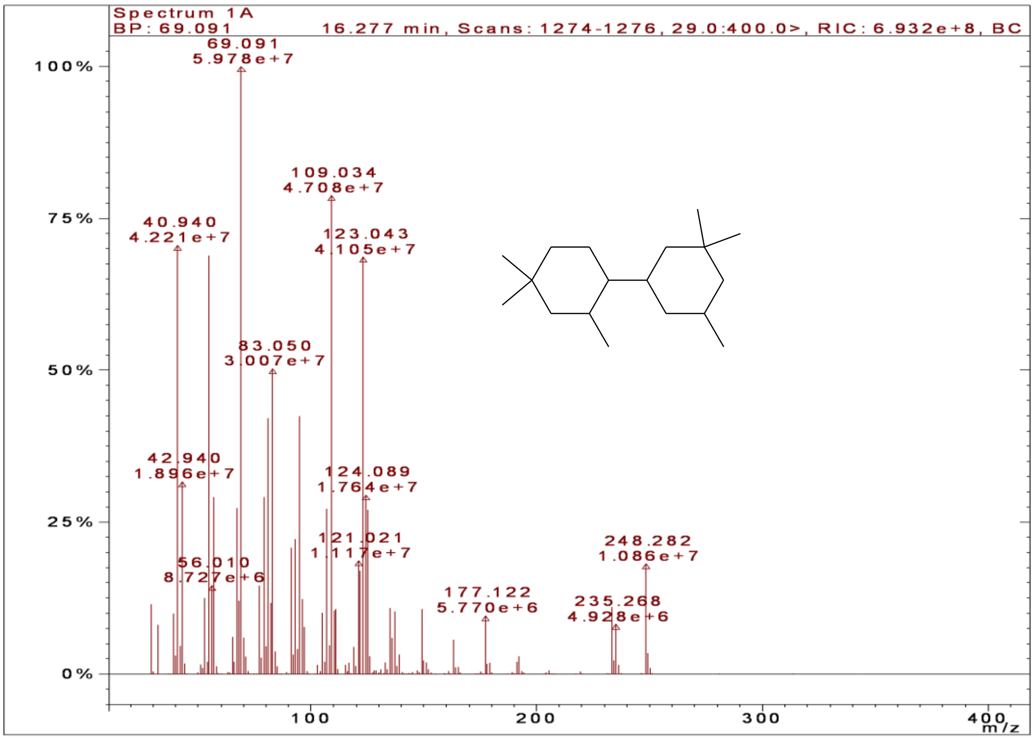

Figure S10 | Mass spectrogram of the 1,1,3-trimethyl-5-(2,4,4-trimethyl-cyclohexyl)cyclohexane from the solvent-free HDO of compound 4.

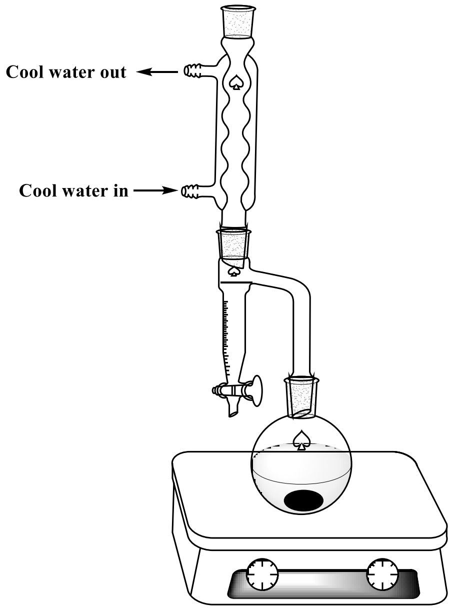

Figure S11 | Reactor for the synthesis of compound 4.

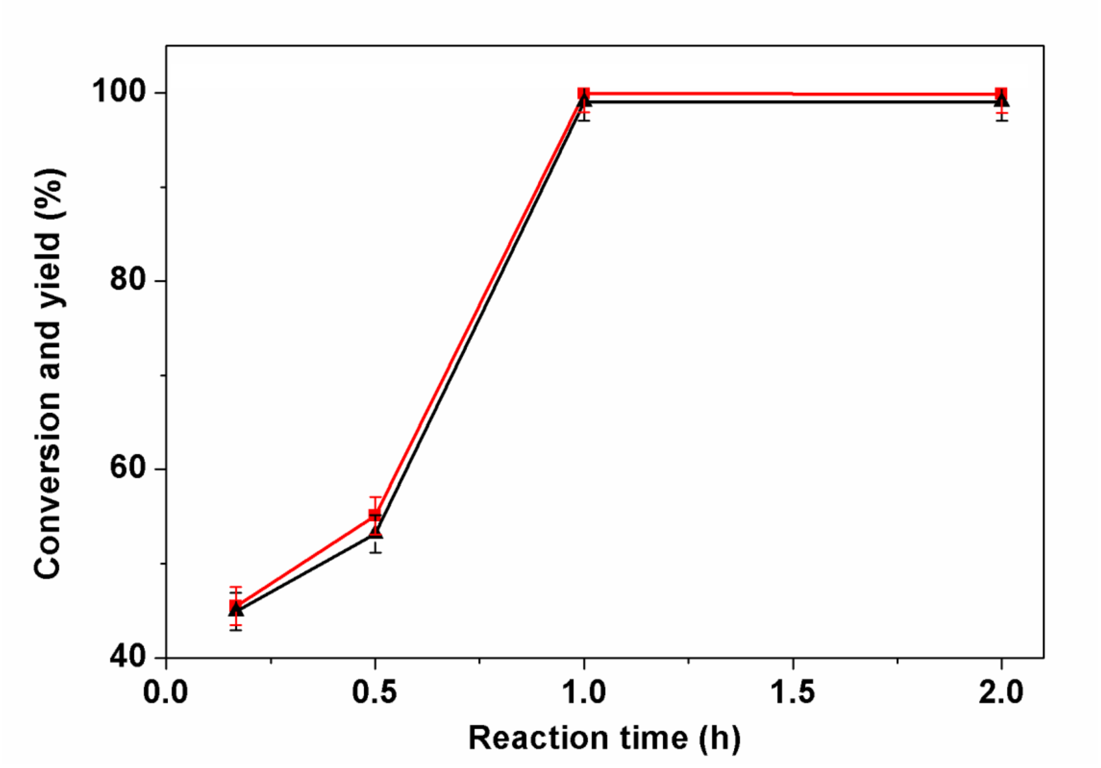

Figure S12 | Isophorone conversions (■) and the carbon yield of compound 2 (▲), over the Pd/C catalyst. Reaction conditions: 298 K, 1 h; 1.16 g (8.4 mmol) isophorone, 0.05 g Pd/C catalyst, 2.0 MPa H_2.

The influence of reaction time on the hydrogenation of isophorone over the Pd/C catalyst was studied. According to Fig. S12, the isophorone conversion and the carbon yields of compound **2** increased synchronously with the increment of reaction time, reached the maximum when reaction was carried out at 298 K for 1 h, then leveled off with the further increasing of reaction time to 2 h. According this result, we fixed the reaction time for the hydrogenation of isophorone as 1 h.

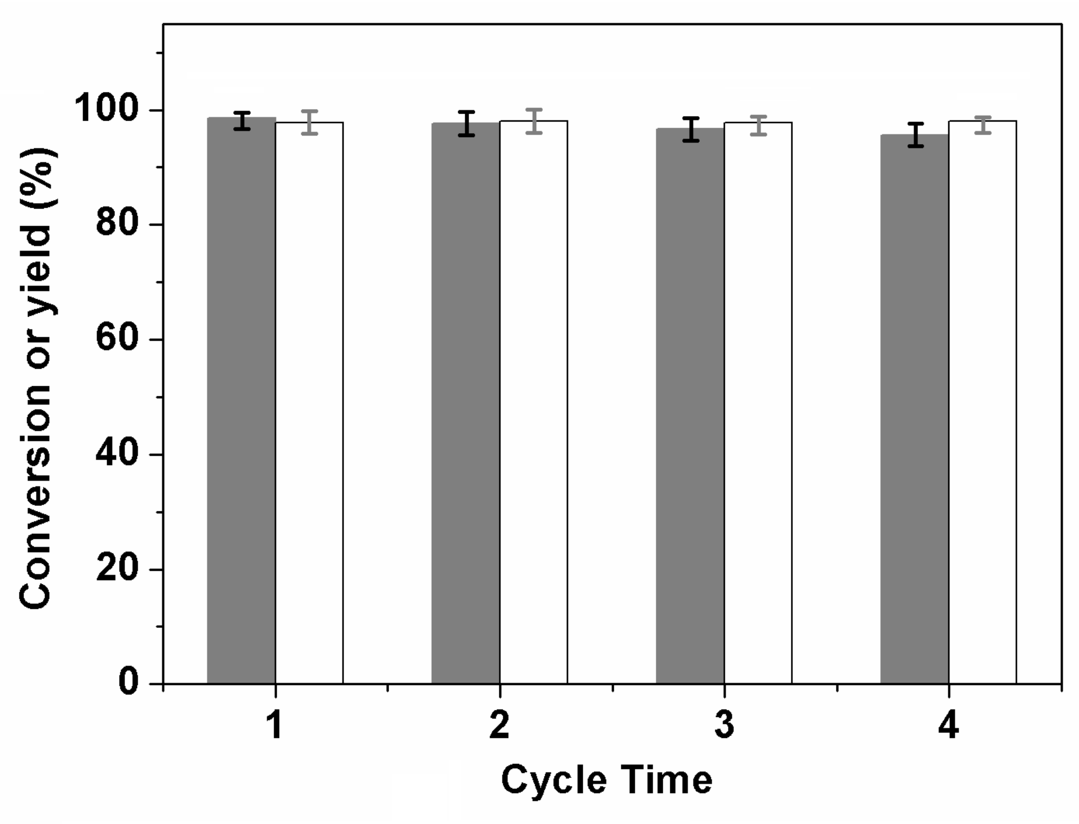

Figure S13 | Isophorone conversions (black bars) and the carbon yield of compound 2 (white bars), over the Pd/C catalyst as the function of recycle time. Reaction conditions: 298 K, 1 h; 1.16 g (8.4 mmol) isophorone, 0.05 g Pd/C catalyst, 2.0 MPa H_2.

The reusability of the Pd/C catalyst was also checked. It was found that this catalyst is stable under the investigated conditions. No evident deactivation was observed during the four repeatedly usages.

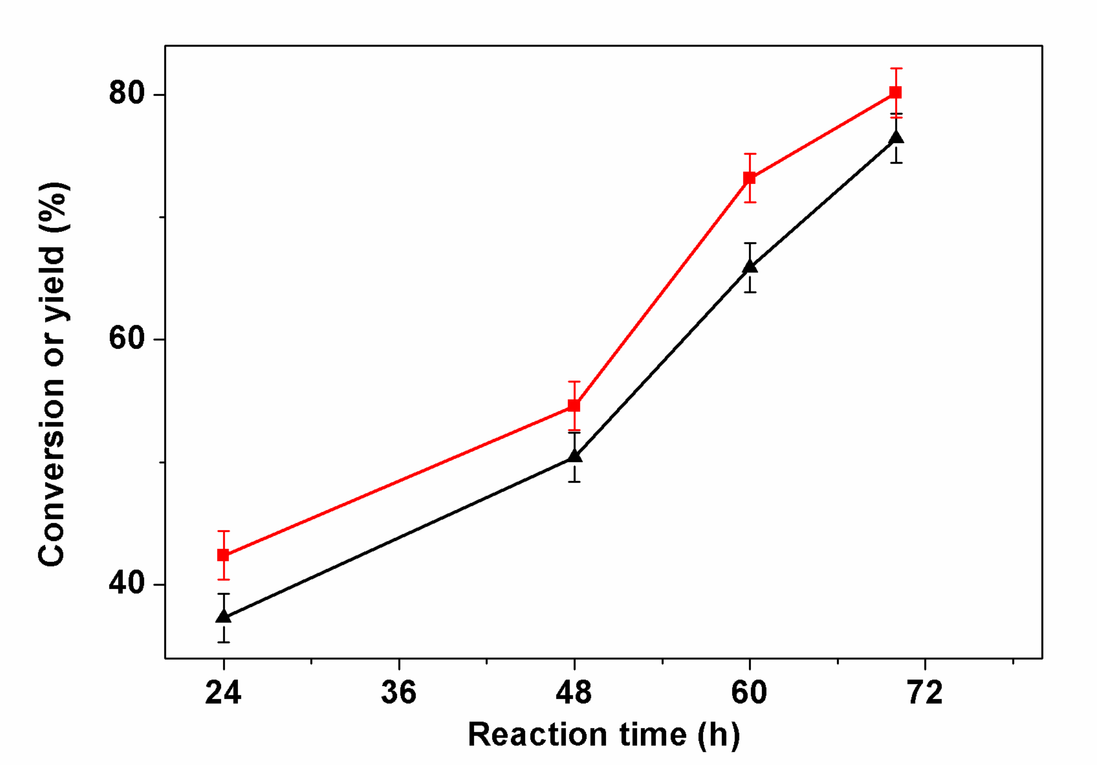

Figure S14 | Conversion of compound 2 (■) and the carbon yield of compound 4 (▲) over NaOH as the function of reaction time. Reaction conditions: 443 K; 20.0 g compound **2**, 50 mmol alkali NaOH and 20 mL *p*-xylene.

The effect of reaction time on the self-aldol condensation of compound **2** over the NaOH catalyst was studied. From Fig. S14, we can see that the conversion of compound **2** and the carbon yield of compound **4** over NaOH catalyst increased with reaction time and reached the maximum (80.1% and 76.4%) when reaction were carried out at 443 K for 72 h. According this result, we fixed the reaction time for the self-aldol condensation of compound **2** as 72 h.

References

1. Huber, G. W., Iborra, S. & Corma, A. Synthesis of transportation fuels from biomass: Chemistry, catalysts, and engineering. *Chem. Rev.* **106**, 4044-4098 (2006).
2. Li, C., Zheng, M., Wang, A. & Zhang, T. One-pot catalytic hydrocracking of raw woody biomass into chemicals over supported carbide catalysts: simultaneous conversion of cellulose, hemicellulose and lignin. *Energy Environ. Sci.* **5**, 6383-6390 (2012).
3. Striebich, R. C. & Lawrence, J. Thermal decomposition of high-energy density materials at high pressure and temperature. *J. Anal. Appl. Pyrol.* **70**, 339-352 (2003).
4. Zhao, C., Camaioni, D. M. & Lercher, J. A. Selective catalytic hydroalkylation and deoxygenation of substituted phenols to bicycloalkanes. *J. Catal.* **288**, 92-103 (2012).
5. Yang, Y., *et al.* Conversion of furfural into cyclopentanone over Ni-Cu bimetallic catalysts. *Green Chem.* **15**, 1932-1940 (2013).
